# Childhood Cancer Patterns in Iran: Challenges and Future Directions

**Published:** 2017-08

**Authors:** Salman KHAZAEI, Somayeh KHAZAEI, Kamyar MANSORI, Erfan AYUBI

**Affiliations:** 1. Dept. of Epidemiology, School of Public Health, Hamadan University of Medical Sciences, Hamadan, Iran; 2. Dept. of Para-Medicine, Rafsanjan University of Medical Sciences, Rafsanjan, Iran; 3. Dept. of Epidemiology, School of Medicine, Kurdistan University of Medical Sciences, Sanandaj, Iran; 4. Dept. of Epidemiology, School of Public Health, Shahid Beheshti University of Medical Sciences, Tehran, Iran

## Dear Editor-in-Chief

Cancers are the second leading cause of death among children 5 to 14 yr, after accidents (1). Although nearly 175000 cancer cases are diagnosed in children younger than 15 yr, unfortunately, fewer than 40% of childhood cancers are diagnosed or receive early and curative treatment (2). Delay in early diagnosis is as result of similarity in signs and symptoms of childhood cancers and common disease in young children (3).

**Fig. 1:**
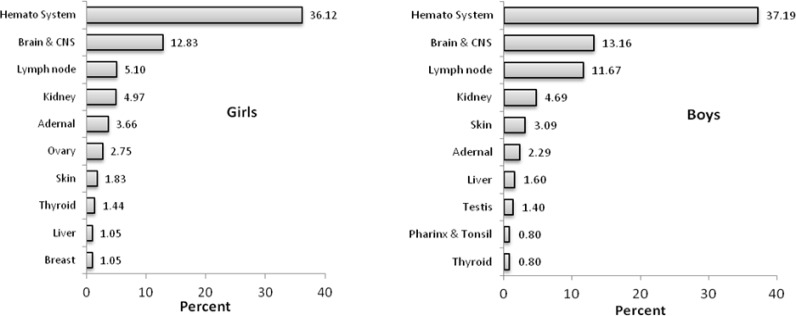
Common cancers among Iranian children, 2008

The overall incidence of childhood cancer in Tehran metropolitan area was 176 per 1000000 (4). Fig. 1 shows 10 common cancers between both genders in Iran. Statistics from reliable sources indicate that totally 764 and 1003 cancer incidence occurred in girls and boys respectively, in 2008. Cancer in hematologic and Brain or central nervous system (CNS) is accountable for nearly half of cancer incidence in both genders in children (5).

In following former pivotal evidence about childhood cancer epidemiology, some reported registry-based studies and regional surveys shows finding as follow: increasing trend in thyroid cancer in all age group except children patients (6), the increasing trend of both brain and spinal tumors in both gender with most incidence in patients aged 1–4 yr (7).

Some evidence from other regions was as follow: the relative percentage of the lymph nodes and skin cancer in Western countries in boys was nearly twice of figures in girls (8). In the United States, common childhood cancers were acute lymphoblastic leukemia (ALL) (26%), brain and CNS (21%), neuroblastoma (7%) in 2014 (9).

In contrast to cancers in adults, there are more challenges and pitfalls in childhood cancers. Small percentage of them has known preventable and modifiable risk factors. Radiation exposure is well established as a cause of childhood cancer because more than half of century it is identified that an association between exposure to medical radiation in pregnancy and risk of leukemia in offspring (10). Constraints in using computed tomography scans health care settings in children and pregnant women were outcomes of archived evidence in this context. In another hand many literatures underpins the association between parental smoking and cancers during childhood (8). Advances in the treatment procedures of childhood cancer in recent decades neutralized with, unknown aspects in etiology and prevention levels of most childhood cancers. In view of methodology, most childhood cancer studies have been conducted in case-control designs that are prone to many biases and also most of them did not examine histologic or molecular subtypes of tumors (9).

According to scare evidence about childhood cancer epidemiology, future studies in Iran should provide evidence about etiology, effectiveness of screening tools and treatment modalities and validity and completeness registries in this context.
